# Gut microbial profile analysis by MiSeq sequencing of pancreatic carcinoma patients in China

**DOI:** 10.18632/oncotarget.18820

**Published:** 2017-06-29

**Authors:** Zhigang Ren, Jianwen Jiang, Haiyang Xie, Ang Li, Haifeng Lu, Shaoyan Xu, Lin Zhou, Hua Zhang, Guangying Cui, Xinhua Chen, Yuanxing Liu, Liming Wu, Nan Qin, Ranran Sun, Wei Wang, Lanjuan Li, Weilin Wang, Shusen Zheng

**Affiliations:** ^1^ Department of Hepatobiliary and Pancreatic Surgery, First Affiliated Hospital, School of Medicine, Zhejiang University, Key Laboratory of Combined Multi-organ Transplantation, Ministry of Public Health, Hangzhou 310003, China; ^2^ State Key Laboratory for Diagnosis and Treatment of Infectious Disease, First Affiliated Hospital, School of Medicine, Zhejiang University, Hangzhou 310003, China; ^3^ Collaborative Innovation Center for Diagnosis and Treatment of Infectious Diseases, Zhejiang University, Hangzhou 310003, China; ^4^ Department of Infectious Diseases, Precision Medicine Center, The First Affiliated Hospital of Zhengzhou University, Zhengzhou 450052, China

**Keywords:** pancreatic carcinoma, gut microbiota, MiSeq sequencing, alpha diversity, biomarkers

## Abstract

Pancreatic carcinoma (PC) is a lethal cancer. Gut microbiota is associated with some risk factors of PC, e.g. obesity and types II diabetes. However, the specific gut microbial profile in clinical PC in China has never been reported. This prospective study collected 85 PC and 57 matched healthy controls (HC) to analyze microbial characteristics by MiSeq sequencing. The results showed that gut microbial diversity was decreased in PC with an unique microbial profile, which partly attributed to its decrease of alpha diversity. Microbial alterations in PC featured by the increase of certain pathogens and lipopolysaccharides-producing bacteria, and the decrease of probiotics and butyrate-producing bacteria. Microbial community in obstruction cases was separated from the un-obstructed cases. *Streptococcus* was associated with the bile. Furthermore, 23 microbial functions e.g. Leucine and LPS biosynthesis were enriched, while 13 functions were reduced in PC. Importantly, based on 40 genera associated with PC, microbial markers achieves a high classification power with AUC of 0.842. In conclusion, gut microbial profile was unique in PC, providing a microbial marker for non-invasive PC diagnosis.

## INTRODUCTION

Pancreatic carcinoma (PC), an aggressively lethal cancer with a poor prognosis, is a common cancer worldwide, accounting for 216,000 new cases annually and has approximately 23% of 1-year survival and 5% of 5-year survival [1, 2]. There are 46,000 estimated new cases and 39,590 deaths in the United States [3]. It is essential to search for new techniques to improve the diagnosis, prognosis and survival of PC. Therefore, bio-markers that can identify early PC are needed.

Recently, thousands of potential bio-markers in blood and tumors are reported [4, 5], but very few have been validated for clinical use [4–6]. The risk factors of PC include age, tobacco, obesity, chronic pancreatitis, and types II diabetes (T2D) [7, 8].

With more knowledge on microbiota, the role of bacteria in carcinogenesis is being recognized [9, 10]. There are reports on the possible association between PC and bacteria, such as H. pylori [11–13]. The role of human microbiota in PC etiology has been studied [14, 15]. The further association between salivary microbiota and PC or chronic pancreatitis has been confirmed [16, 17].

Gut microbiota works as the biggest micro-ecosystem [18, 19]. It is closely associated with a series of chronic diseases, such as obesity [20], non-alcoholic fatty liver disease [21, 22] and T2D [23]. These diseases are also risk factors for PC [7, 8]. Gut microbiota can also promote carcinogenesis, e.g. hepatocellular carcinoma (HCC) [24] and colorectal cancer [25, 26], and regulate inflammation [27, 28]. Thus, it is hypothesized that gut microbiota is associated with PC but gut microbial characteristics in clinical PC have not been reported. In this prospective study, 167 stool samples were collected from patients with pancreatic neoplasm and healthy controls (HC). After confirmation exams, 85 PC patients and 57 matched HC were processed for Miseq sequencing. The gut microbial composition, taxonomic difference, microbial function prediction and microbial markers were performed.

## RESULTS

**Figure 1:**
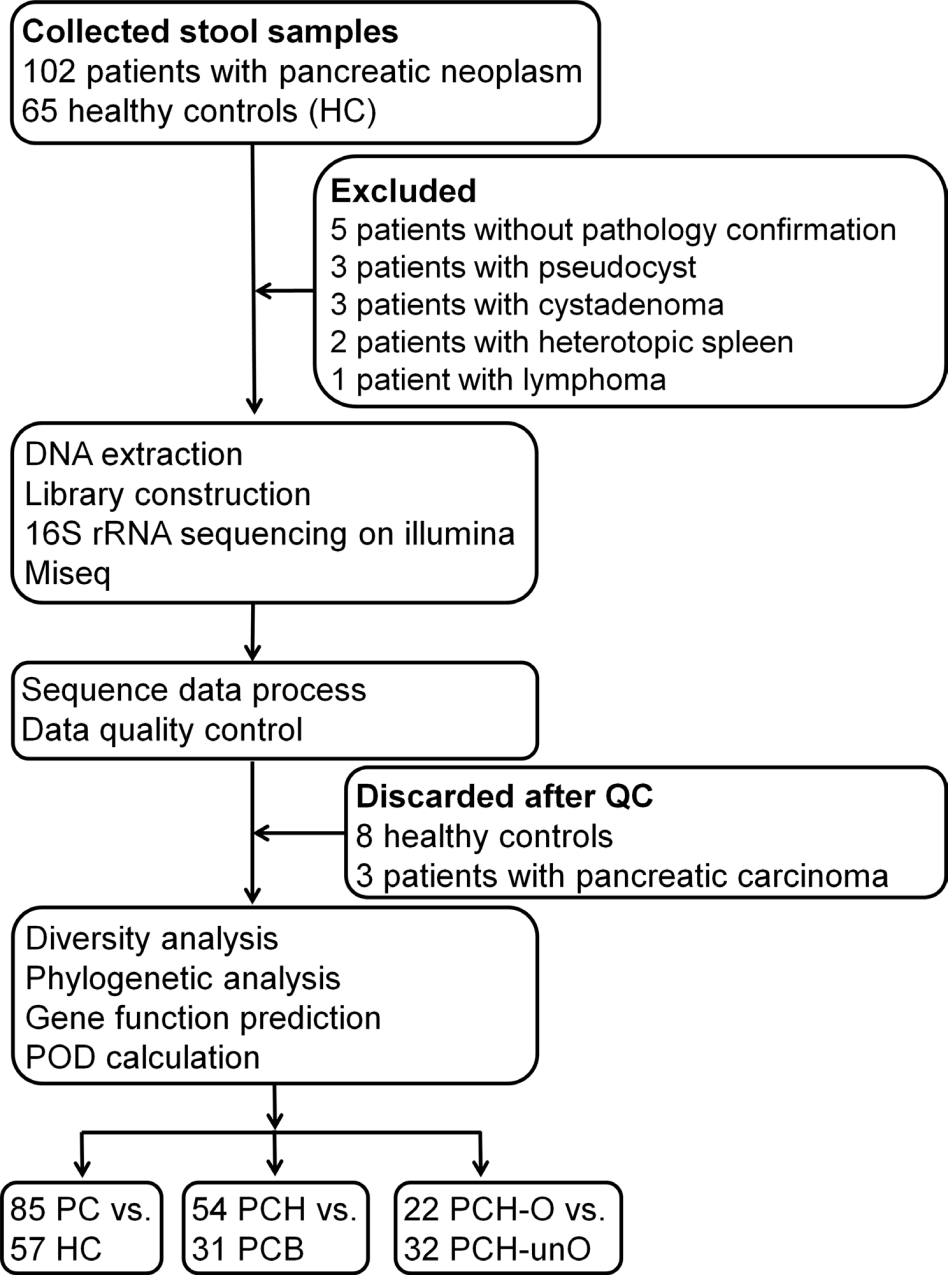
Study design and flow diagram A total of 167 fecal samples from 102 patients with pancreatic neoplasm and 65 healthy controls were collected. After a strict pathologic diagnosis and exclusion process, the remained samples were used for DNA extraction, 16S rRNA sequencing and data quality control. Finally, 85 patients with PC and 57 healthy controls were utilized for bioinformatics analysis. Meanwhile, subgroup analysis and stratification analysis for PC were performed. BMI, body mass index; QC, quality control; POD, probability of disease; HC, healthy controls; PC, pancreatic carcinoma; PCH, PC (head); PCB, PC (body and tail); PCH-O, PCH with obstruction of common bile duct; PCH-unO, PCH with unobstruction of common bile duct.

A total of 102 patients with pancreatic neoplasm and 65 HC with matched age, gender and body mass index (BMI) were enrolled initially. After confirmation, 5 patients without pathology confirmation, 3 patients with pseudo-cyst, 3 patients with cystadenoma, 2 patients with hetero-topic spleen and 1 patient with lymphoma were excluded. After DNA extraction, 16S rRNA sequencing and data quality control, 8 HC and 3 PC patients were further discarded. Finally, 85 PC and 57 matched HC samples were included for the final analysis (Figure 1). According to the anatomy of PC, 85 PC were divided into 54 (63.5%) pancreatic head cancers (PCH) and 31 (36.5%) body and tail cancers (PCB); according to serum levels of direct bilirubin (DB), 54 PCH were divided into 22 (40.7%) PCH with obstruction of common bile duct (PCH-O) and 32 (59.3%) PCH with the un-obstruction (PCH-unO). Based on clinical TNM staging, 54 cases of PC were stage I and 31 cases were stage II. As for PCH, 32 cases of PCH were stage I and 22 cases were stage II, which were consistent with the un-obstruction and obstruction classification.

As shown in Table 1, there were no significant differences in age, gender and BMI between PC and HC. In clinical parameters, PC patients presented increased levels of DB and tumor markers, but a decreased level of albumin, as compared with HC. PCH patients showed elevated levels of alanine transaminase (ALT), aspartate aminotransferase (AST), total bilirubin and DB, versus PCB patients. Notably, the indicators in PCH were mainly attributed to PCH-O patients who presented significantly increased ALT, AST, gamma-glutamyl transpeptidase, total bilirubin and DB, versus PCH-unO.

**Table 1:** Matched clinical information in PC patients and healthy controls

	PC (*N* = 85)	HC (*N* = 57)	*P* value	PCH (*N* = 54)	PCB (*N* = 31)	*P* value	PCH-O (*N* = 22)	PCH-unO (*N* = 32)	*P* value
*Age, median years (min-max)	56 (33–78)	52 (43–67)	0.08	56 (33–78)	56 (38–68)	0.23	59.5 (45–78)	54.5 (33–70)	0.22
# Gender, male (%)	47 (55.3)	36 (63.2)	0.39	28 (51.9)	19 (61.3)	0.50	15 (0.68)	13 (40.6)	0.06
BMI, median kg/m^2^(min-max)	22.7 (19.5–26.0)	23.2 (18.5–27.1)	0.06	22.8 (20.4–24.6)	22.5 (19.5–26.0)	0.08	22.9 (20.7–24.6)	22.6 (20.4–24.2)	0.23
ALT, U/L median (min-max)	20 (5–525)	21 (8–41)	0.36	33.5 (7–525)	15 (5–57)	< 0.01	67 (14–525)	19.5 (7–55)	< 0.01
AST, U/L median (min-max)	23 (10–292)	22 (12–40)	0.08	32.5 (14–292)	18 (10–77)	< 0.01	56 (22–292)	21.5 (14–80)	< 0.01
Clinical TNM staging	I: 54 II: 31			I: 32 II: 22	I: 22 II: 9		I: 0 II: 22	I: 32 II: 0	
Albumin, g/L, median (min-max)	40.6 (27.6–406)	48.6 (21–53.4)	< 0.01	40.5 (27.6–48)	41.4 (30.6–406)	0.07	39.7 (27.6–48)	41 (31.1–46.2)	0.27
Globulin, g/L, median (min-max)	26.7 (16.9–37)	26 (20.3–33.3)	0.75	27.0 (17.9–37)	25.3 (16.9–36.6)	0.44	27.2 (17.9–37)	27.0 (18.1–36)	0.69
Total bilirubin, μmol/L, median (min-max)	14 (4–389)	12 (6–26)	0.11	16.5 (4–389)	10.5 (6–36)	< 0.01	143.5 (17–389)	11 (4–17)	< 0.01
Direct bilirubin, μmol/L, median (min-max)	5 (2–277)	4 (2–10)	< 0.01	6 (2–277)	4 (2–7)	< 0.01	99 (16–277)	4.5 (2–7)	< 0.01
CEA ng/mL, median (min-max)	2.7 (0.8–178)	1.8 (0.5–24)	< 0.01	2.8 (0.8–178)	2 (0.8–26.3)	0.23	3.9 (1.6–14.4)	2.0 (0.8–178.1)	0.03
CA 199, U/mL, median (min-max)	34.8 (2–12000)	8.35 (2–50.3)	< 0.01	35.5 (2–8130)	14 (2–12000)	0.12	35.5 (3.8–2372)	35.4 (2–8130.4)	0.57
CA 125, U/mL, median (min-max)	17.2 (4.3–362.7)	8.8 (3.2–30.5)	< 0.01	17.5 (4.3–362.7)	15.1 (4.7–185.5)	0.84	22.5 (7.5–362.7)	14.1 (4.3–96.6)	0.15

BMI, body mass index; ALT, alanine transaminase; AST, aspartate aminotransferase; CEA, carcinoembryonic antigen; CA 199, carbohydrate antigen 199; CA 125, carbohydrate antigen 125; PC, pancreatic carcinoma; HC, healthy controls; PCH, pancreatic head cancer; PCB, pancreatic body and tail cancer; PCH-O, PCH with obstruction of bile duct; PCH-unO, PCH with unobstruction of bile duct. *Continuous variables was compared by Wilcoxon rank sum test; #Categorical variables was compared by Fisher's exact test.

The final 142 samples from PC and HC were pooled into 14 libraries according to 16S rRNA sequencing data. The 4,816,686 qualified reads from 9,179,689 raw reads were filtered for downstream analysis. The 2,130,000 reads were chosen randomly from each sample with a 15,000 reads cutoff. Finally, 880 Operational Taxonomy Units (OTUs) were obtained and annotated (Supplementary Data File 1), in which 535 qualified OTUs were clustered (Supplementary Data File 2), but 345 OTUs were discarded because of their low coverage. The 4,602,883 (95.56%) of all qualified reads could be clustered into qualified OTUs with randomly chosen qualified reads. Notably, 98.0665% and 92.3249% of all reads were assigned into family and genus levels respectively.

### Gut microbial diversity is decreased in PC

**Figure 2:**
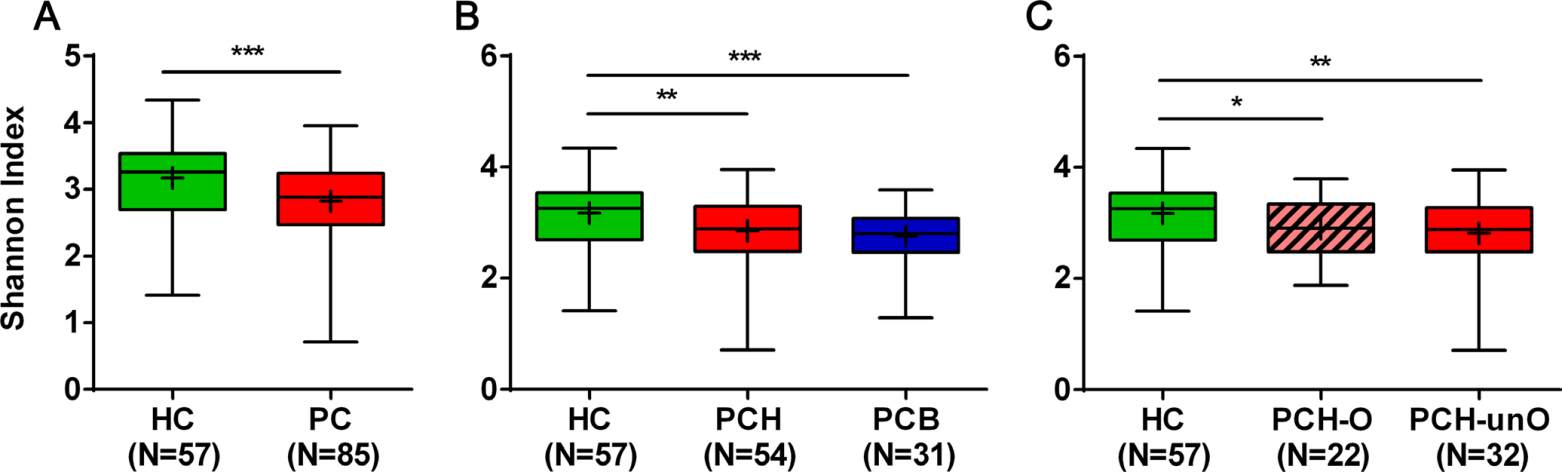
Microbial alpha diversity decreased in PC patients shown by Shannon diversity index (**A**) The comparison of Shannon diversity index between PC patients (*n* = 85) and HC (*n* = 57). (**B**) The subgroup comparison of Shannon diversity index among HC (*n* = 57), PCH (*n* = 54) and PCB (*n* = 31). (**C**) The stratification analysis of Shannon diversity index among HC (*n* = 57), PCH-O (*n* = 22) and PCH-unO (*n* = 32). The box presents the interquartile range; the line inside denotes the median, and the symbol “+” denotes the mean value. HC, healthy controls; PC, pancreatic carcinoma; PCH, PC (head); PCB, PC (body and tail); PCH-O, PCH with obstruction of common bile duct; PCH-unO, PCH with unobstruction of common bile duct. *p < 0.05, **p < 0.01, ***p < 0.001.

Gut microbial diversity was analyzed after equalizing sample sizes to 15,000 reads by random subtraction. Compared with HC, microbial diversity significantly decreased in PC, as estimated by Shannon index (2.82 vs 3.17, *p <* 0.001) with two tailed unpaired t test (Figure 2A and Supplementary Data File 3). This was also validated by other diversity parameters (Chao1: 195.8 vs 174.6, *p <* 0.01 and Simpson: 0.89 vs 0.85, *p <* 0.01, Supplementary Figure 1A). The subgroup analysis of PC indicated that alpha diversity index, shown by Shannon index, was remarkably reduced in both PCH and PCB as compared with HC (both *p <* 0.01). No obvious difference was observed between PCH and PCB (Figure 2B). For further stratification analysis of PCH, alpha diversity index decreased in both PCH-O and PCH-unO versus HC (both *p <* 0.05), whereas there was no statistical difference between PCH-O and PCH-unO (Figure 2C). It was verified by Chao 1 and Simpson indices (Supplementary Figure 1B and 1C).

### Gut microbial profile is unique in PC

**Figure 3:**
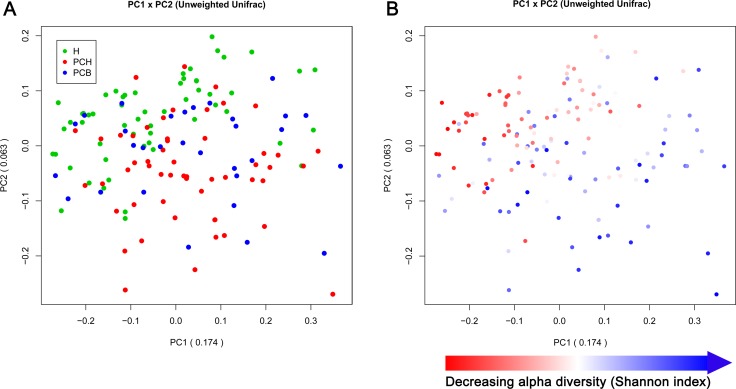
Principal coordinate analysis (PCoA) based on the unweighted Unifrac metric of fecal microbiota among all samples (**A**) Gut microbial profile in patients with PC was significantly distinct from HC (*p <* 0.001), but no remarkable separation was observed between PCH and PCB patients, shown by PCoA analysis. (**B**) The same PCoA plot as in (A), but the samples were colored according to their Shannon diversity indices, suggesting alpha diversity as an important factor along PC 1. PC, principal component; H, healthy controls; PCH, pancreatic carcinoma (head); PCB, pancreatic carcinoma (body and tail).

To illustrate similarity of different samples in bacterial communities, the principal coordinate analysis (PCoA) based on OTUs distribution was conducted. On the unweighted Unifrac plot, fecal microbial communities separated between PC and HC from principal component (PC) 1 and PC2 (17.4% and 6.3% of explained variance, respectively, *p <* 0.001), suggesting an unique gut microbiota in PC, while no obvious separation was observed between PCH and PCB (Figure 3A). Notably, alpha diversity, measured by Shannon index, could be identified as one of the main factors contributing to the separation and difference of gut microbiota between PC and HC along PC1 (Figure 3B).

The subgroup analysis of PC based on PCoA demonstrated that microbial community presented no significant difference between PCH and PCB in the unweighted Unifrac plot from PC1 and PC2 (18.5% and 6.7%) and weighted Unifrac plot from PC1 and PC2 (41.7% and 10.6%) (Supplementary Figure 2A and 2B). Furthermore, the stratification analysis of PCH based on PCoA indicated that microbial community in PCH-O were clustered together and significantly separated from PCH-unO in the unweighted Unifrac plot from PC1 and PC2 (25.7% and 20.7%, *p <* 0.001) and weighted Unifrac plot from PC1 and PC2 (59.5% and 12.4%, *p <* 0.001) (Supplementary Figure 2C and 2D).

### Phylogenetic profiles of gut microbiota in PC

**Figure 4:**
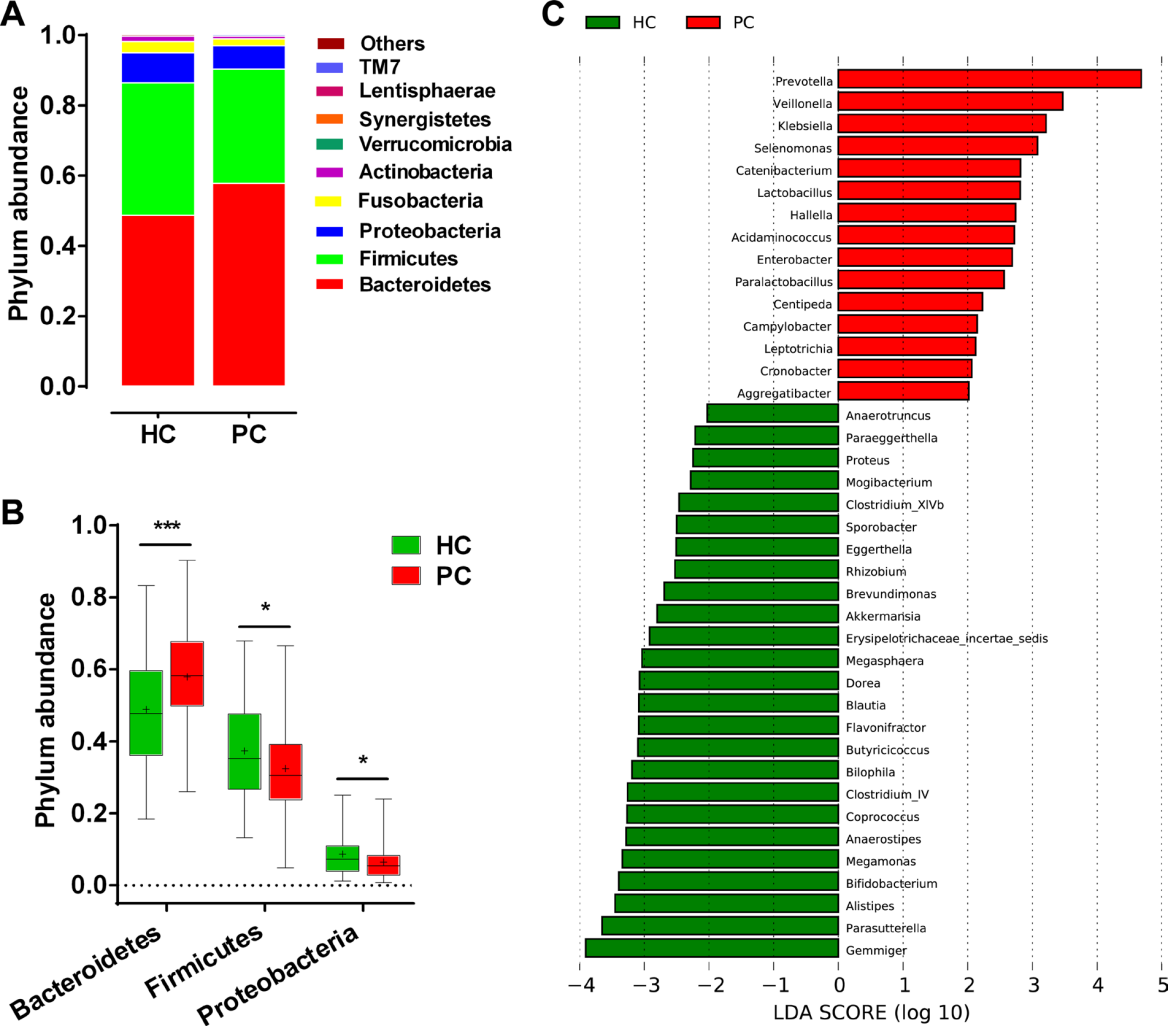
Composition and difference of fecal microbial communities between PC patients (*n* = 85) and healthy controls (*n* = 57) (**A**) Composition of fecal microbiota at the phylum level between the two groups. (**B**) Phylum *Bacteroidetes* was significantly increased, while *Firmicutes* and *Proteobacteria* were decreased in PC patients versus healthy controls. The box presents the interquartile range; the line inside denotes the median, and the symbol “+” denotes the mean value. (**C**) The 40 genera were significantly different between PC patients and healthy controls, shown by LDA score (log 10). The 15 genera were increased, whereas 25 were decreased in PC patients versus healthy controls. LDA, linear discriminant analysis; HC, healthy controls; PC, pancreatic carcinoma.

The bacterial taxonomic composition and alterations in PC was analyzed. Phylum abundance and composition were shown in Supplementary Figure 3A. *Bacteroidetes*, *Firmicutes* and *Proteobacteria* were the dominant bacterial phyla in PC and HC (Figure 4A). Compared with HC, *Bacteroidetes* significantly increased (*p <* 0.001), while *Firmicutes* and *Proteobacteria* decreased in PC (both *p <* 0.05) (Figure 4B). Correspondingly, genus abundance and composition were listed the top 19 genera in both groups (Supplementary Figure 3B). The linear discriminant analysis (LDA) effect size (LEfSe) method was utilized to select the greatest differences in taxa between PC and HC. A representative cladogram of fecal microbial structure indicated a significant gut microbial imbalance in PC (Supplementary Figure 4). At the genus level, 15 taxa mainly including *Prevotella*, *Veillonella*, *Klebsiella*, *Selenomonas*, *Hallella*, *Enterobacter* and *Cronobacter* were significantly enriched, while 25 taxa mainly including *Gemmiger*, *Bifidobacterium*, *Coprococcus*, *Clostridium IV*, *Blautia*, *Flavonifractor*, *Anaerostipes*, *Butyricicoccus* and *Dorea* were remarkably reduced in PC versus HC based on LDA selection (Figure 4C). These results suggest that gut microbial alterations in PC present significant increase of some potential pathogens and lipopolysaccharides (LPS)-producing bacteria, and obvious decrease of some probiotics and butyrate-producing bacteria.

### Crucial bacteria associated with the bile in PC

**Figure 5:**
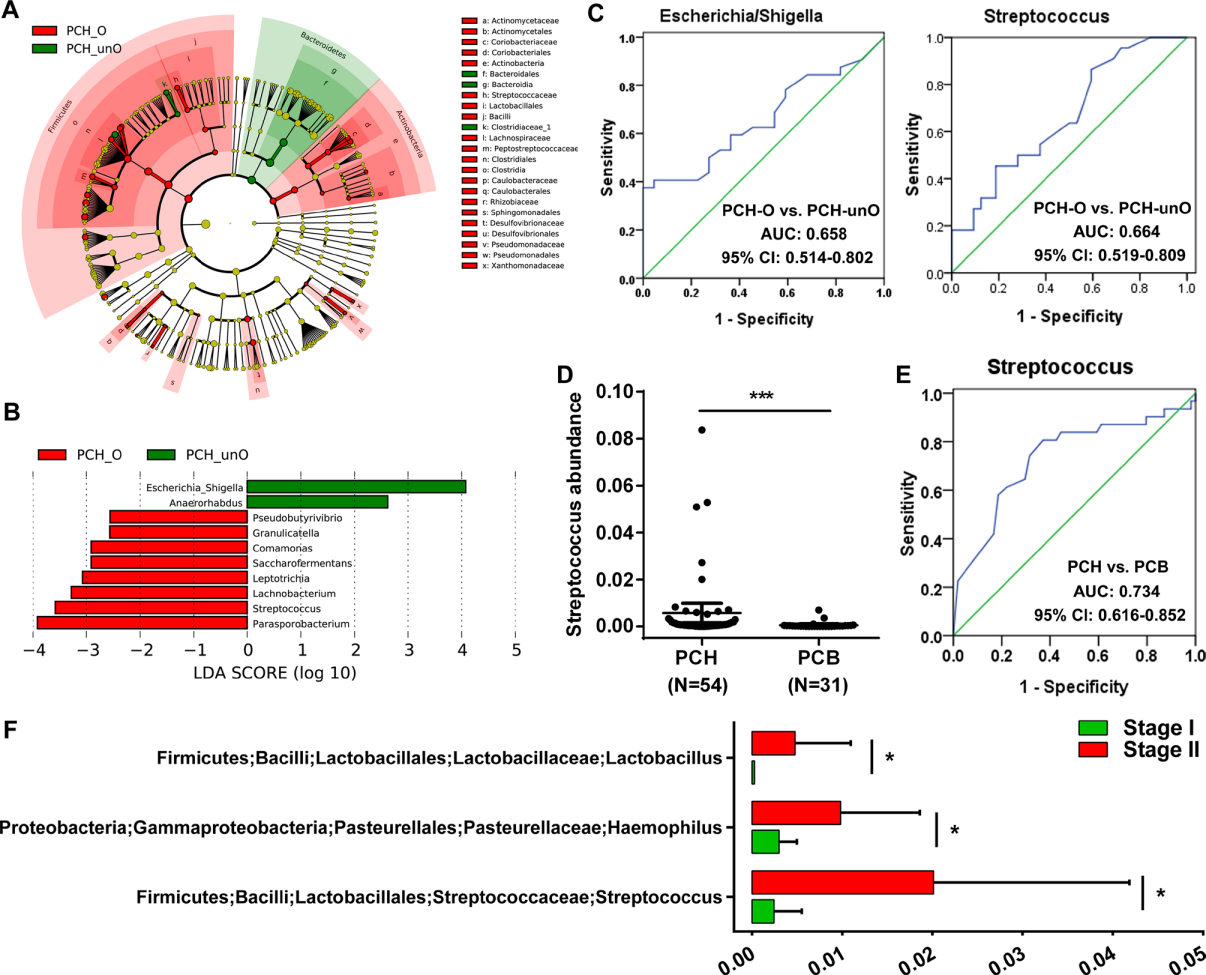
Identification of crucial bacteria associated with the bile in the gut for PC (**A**) Phylogenetic profiles of the specific bacterial taxa and predominant bacteria associated with PCH-O using the LEfSe method. (**B**) The greatest differences at the genus level between PCH-O and PCH-unO, shown by LDA score (log 10). LDA, linear discriminant analysis. (**C**) Abundance of genus *Streptococcus* was significantly increased in PCH (*N* = 54) versus PCB (*N* = 31) patients. (**D**) Abundance of genus *Streptococcus* was elevated in PCH-O (*N* = 22) versus PCH-unO (*N* = 32) patients. PC, pancreatic carcinoma; PCH, PC (head); PCB, PC (body and tail); PCH-O, PCH with obstruction of common bile duct; PCH-unO, PCH with unobstruction of common bile duct. (**E**) The abundance of *Streptococcus* might distinguish PCH from PCB, achieving an AUC of 0.734 (95% CI: 0.616–0.852). (**F**) Genera *Lactobacillus*, *Haemophilus* and *Streptococcus* were significantly enriched in Stage II versus Stage I PC patients.

The obstruction of common bile duct in PC led to bile deficiency in the gut, and the bile was an important factor affecting gut microbial status. Gut microbial community in PCH-O was significantly different from PCH-unO. LEfSe method was used to display the greatest difference of microbial structure between PCH-O and PCH-unO (Figure 5A). At the genus level, 8 taxa mainly including *Parasporobacterium* and *Streptococcus* enriched, whereas *Escherichia Shigella* and *Anaerorhabdus* reduced in PCH-O versus PCH-unO based on LDA selection (Figure 5B). To some extent, abundances of *Streptococcus* and *Escherichia Shigella* might distinguish PCH-O from PCH-unO, and their area under the curve (AUC) values were 0.664 (95% confidence interval (CI): 0.519–0.809) and 0.658 (95% CI: 0.514–0.802), respectively (Figure 5C). Thus, *Streptococcus* and *Escherichia Shigella* may be associated with the bile in PC.

Gut microbial difference between PCH and PCB was mainly derived from the part of PCH-O. Then, microbial taxonomic difference between PCH and PCB was compared (Supplementary Figure 5). Notably, *Streptococcus* abundance significantly increased in PCH versus PCB (*p <* 0.001, Figure 5D). Meanwhile, the abundance of *Streptococcus* might distinguish PCH from PCB, achieving an AUC of 0.734 (95% CI: 0.616–0.852) (Figure 5E). These results were consistent with the difference between PCH-O and PCH-unO, suggesting that *Streptococcus* is closely associated with the bile in PC.

According to international TNM stage of PC, a total of 50 PC patients were assigned to Stage I and 25 PC patients were assigned to Stage II. To further illustrate gut microbial changes along PC progression, we analyzed microbial difference between Stage I and Stage II. The results indicated that genera *Lactobacillus*, *Haemophilus* and *Streptococcus* were significantly enriched in Stage II versus Stage I PC patients (Figure 5F), which might provide novel therapeutic targets to prevent PC progression.

### Functional prediction of microbial gene associated with PC

**Figure 6:**
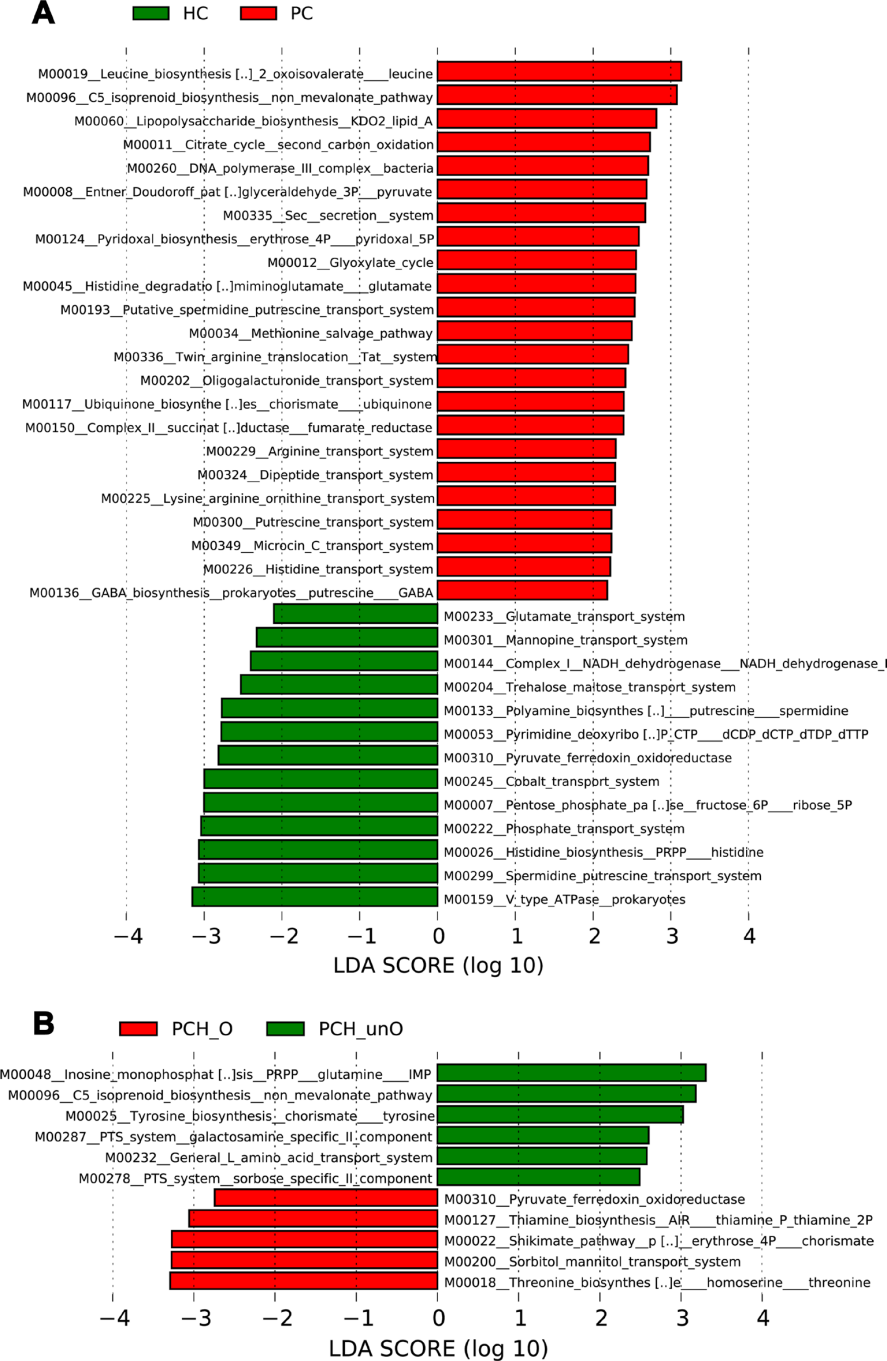
Functional prediction of microbial genes associated with PC using PICRUSt (**A**) The 23 predicted microbial functions including Leucine biosynthesis, Isoprenoid biosynthesis non mevalonate pathway and LPS biosynthesis were significantly enriched, while 13 functions including Type V ATPase in prokaryotes, Spermidine putrescine transport system and Histidine biosynthesis were remarkably reduced in PC patients versus HC, shown by LDA score (log 10). (**B**) The 6 predicted microbial functions including Inosine monophosphate biosynthesis, C5 isoprenoid biosynthesis non mevalonate pathway and Tyrosine biosynthesis were significantly increased, whereas 5 functions including Threonine biosynthesis, Sorbitol mannitol transport system and Shikimate pathway of phosphoenolpyruvate were decreased in PCH-unO versus PCH-O patients, shown by LDA score (log 10). PICRUSt: Phylogenetic Investigation of Communities by Reconstruction of Unobserved States; LDA, linear discriminant analysis; HC, healthy controls; PC, pancreatic carcinoma; PCH, PC (head); PCH-O, PCH with obstruction of common bile duct; PCH-unO, PCH with unobstruction of common bile duct.

Phylogeny and function of gut microbial community are linked. The phylogenetic investigation of communities by reconstruction of unobserved states (PICRUSt) version 1.0.0 pipeline [29] and human version 0.99 [30] were used to construct KEGG pathway/module profile and predict functional capacity of microbial communities using 16S rRNA marker gene sequences (Supplementary Data File 4). Based on LDA selection, compared with HC, 23 predicted microbial functions including Leucine biosynthesis, Isoprenoid biosynthesis non mevalonate pathway and LPS biosynthesis enriched, while 13 functions including Type V ATPase in prokaryotes, Spermidine putrescine transport system and Histidine biosynthesis reduced in PC (Supplementary Data File 5, Figure 6A). Moreover, compared to PCH-O, 6 functions including Inosine monophosphate biosynthesis, C5 isoprenoid biosynthesis non mevalonate pathway and Tyrosine biosynthesis increased, whereas 5 functions including Threonine biosynthesis, Sorbitol mannitol transport system and Shikimate pathway of phosphoenolpyruvate decreased in PCH-unO (Supplementary Data File 6, Figure 6B), suggesting that these microbial gene functions are closely associated with the bile in the gut.

### Classification power of microbial markers associated with PC

**Figure 7:**
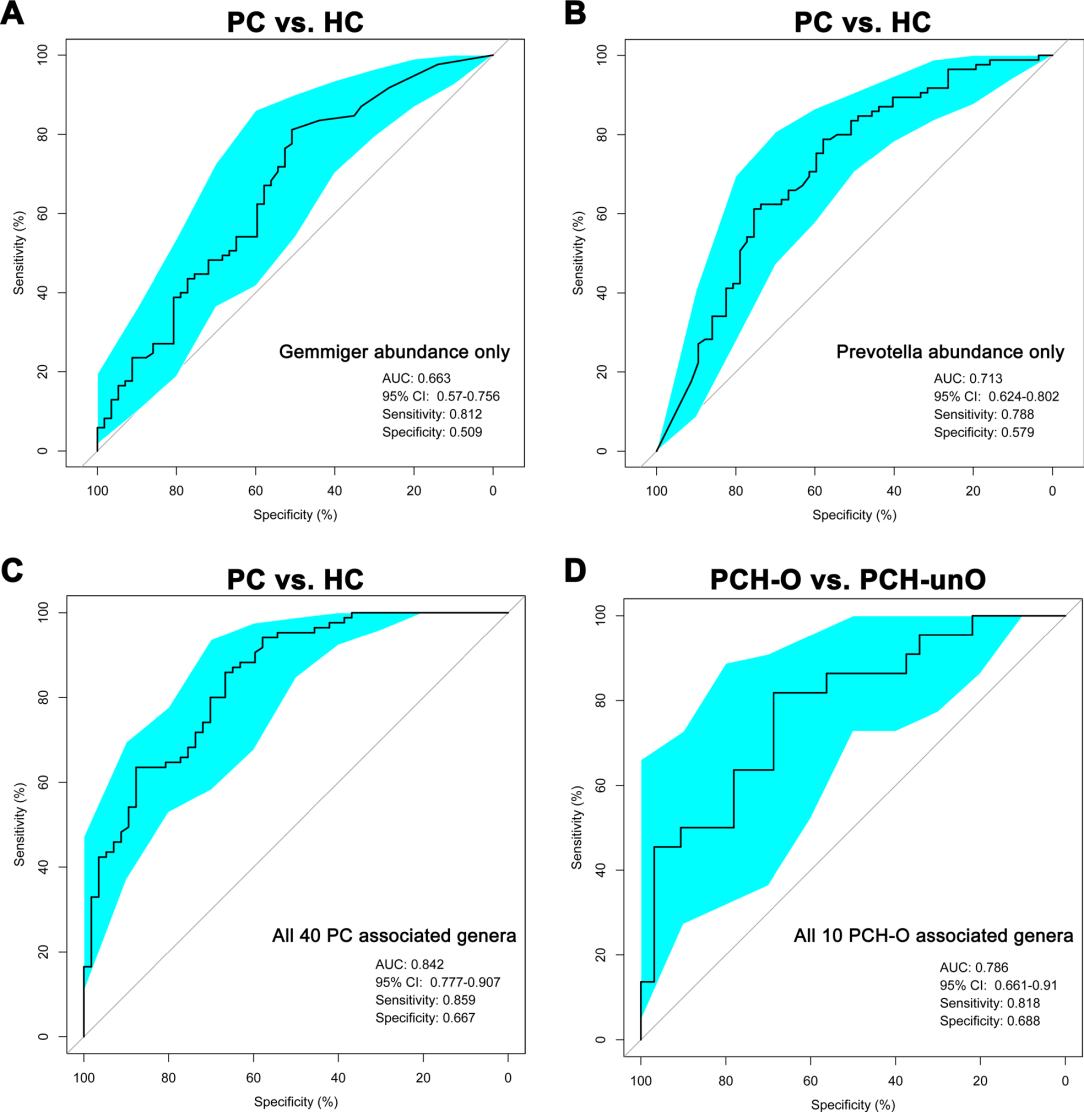
Classification power of microbial markers associated with PC by AUROC analysis (**A**) The abundance of *Gemmiger* was used to distinguish PC patients from healthy controls (AUC = 0.663, 95% CI: 0.57–0.756, sensitivity: 0.812, specificity: 0.509). (**B**) The abundance of *Prevotella* was utilized to distinguish PC patients from healthy controls (AUC = 0.713, 95% CI: 0.624–0.802, sensitivity: 0.788, specificity: 0.579). (**C**) All 40 PC associated genera were transformed as probability of disease (POD) using R package “randomForest”, and then were used to distinguish PC patients from healthy controls shown by AUROC analysis (AUC = 0.842, 95% CI: 0.777–0.907, sensitivity: 0.859, specificity: 0.667). (**D**) All 10 PCH-O associated genera were transformed as POD using R package “randomForest”, and then were used to distinguish PCH-O from PCH-unO patients (AUC = 0.786, 95% CI: 0.661–0.91, sensitivity: 0.818, specificity: 0.688). AUC, area under the curve; AUROC, area under the receiver operating characteristics curve; CI: confidence intervals; PC, pancreatic carcinoma; HC, healthy controls; PCH, PC (head); PCH-O, PCH with obstruction of common bile duct; PCH-unO, PCH with unobstruction of common bile duct.

To explore classification potential of gut microbial markers in PC, area under the receiver operating characteristics curve (AUROC) was performed using the abundance of the bacteria with biggest difference between PC and HC. The abundance of *Gemmiger* only gave an AUC of 0.663 (95% CI: 0.57–0.756) (Figure 7A), while *Prevotella* abundance only achieved an AUC of 0.713 (95% CI: 0.624–0.802) (Figure 7B) between PC and HC.

Furthermore, the 10-fold cross validation model was set up by using R package “randomForest” [31] to calculate probability of disease (POD). Based on the 40 genera associated with PC using LDA selection, the AUC increased to 0.842 (95% CI: 0.777–0.907) between PC and HC (Figure 7C), achieving a high classification power for PC. When we performed 1000 permutations, randomly assigning samples to the training cohort, the AUC of the 40 genera still achieved similar results (Supplementary Figure 6A).

In addition, the same method to calculate POD between PCH-O and PCH-unO was conducted by using the combination of 10 genera associated with PCH-O based on LDA selection. The AUC reached 0.786 (95% CI: 0.661–0.91) between PCH-O and PCH-unO (Figure 7D), presenting a significant increase of classification power for PCH-O. The random assignment of 1000 permutations based on the 10 genera also showed similar results (Supplementary Figure 6B).

## DISCUSSION

Understanding the etiology of PC lead to new prevention or treatment [32]. Some high risk factors including obesity, non-alcoholic fatty liver disease and T2D, have already been found close association with PC development [7, 8], and gut microbiota is proven involved in these diseases [20–23] or promote carcinogenesis e.g. HCC [24] and colorectal cancer [25]. However, gut microbial characterization in human PC have not been reported. This study, for the first time, illustrates gut microbial profile in PC cohorts in China by Miseq sequencing. Gut microbial diversity is significantly decreased and microbial profile is unique in PC, partly attributed to the decrease of alpha diversity. Microbial alterations in PC present an increase of some potential pathogens and LPS-producing bacteria, and a decrease of some probiotics and butyrate-producing bacteria. Moreover, microbial community in PCH-O is separated from PCH-unO, and *Streptococcus* is closely associated with the bile in the gut. Importantly, based on the 40 genera associated with PC, microbial markers achieve a high classification power between PC and HC, providing non-invasive bio-markers for PC diagnosis.

Gut microbial imbalance in chronic diseases including T2D [33], inflammatory bowel diseases [34] and liver cirrhosis [35] are unique for each disease. Different diseases display a relatively unique profile, even if some markers are shared [36]. In T2D, gut microbiota presented a moderate degree of imbalance, characterized by the decrease of some universal butyrate-producing bacteria and the increase of various opportunistic pathogens, as well as the enrichment of other microbial functions conferring sulphate reduction and oxidative stress resistance [33]. In liver cirrhosis, potentially pathogenic bacteria including families *Enterobacteriaceae*, *Veillonellaceae* and *Streptococcaceae* were prevalent, with the reduction of beneficial populations such as *Lachnospiraceae* [35]. In PC of our study, some potential pathogens including *Veillonella*, *Klebsiella* and *Selenomonas* and LPS-producing bacteria including *Prevotella*, *Hallella* and *Enterobacter* were enriched, whereas probiotics including *Bifidobacterium* and some butyrate-producing bacteria, such as *Coprococcus*, *Clostridium IV*, *Blautia*, *Flavonifractor* and *Anaerostipes* were reduced. Importantly, the 40 genera associated with PC achieved an excellent classification capacity with AUC of 0.842 between PC and HC, suggesting that the specific alterations of gut microbiota might become non-invasive bio-markers for PC diagnosis. A successful bio-marker for cancer diagnosis should possess abilities of achieving high accuracy, easily operable and non-invasive specimen, and cost-effectiveness benefit. Fecal sample satisfied the non-invasive and easily operable features. Our study indicated that based on crucial genera associated with PC, gut microbial markers might achieve a high classification power for PC (AUC: 0.842, 95% CI: 0.777–0.907), which indicated the high accuracy feature of gut microbial marker. Also, the cost of microbial Miseq sequencing will be significantly decreased along with the development of high-throughput sequencing. Thus, gut microbiota could be a successful biomarker for cancer diagnosis.

Phylogeny and function of gut microbiota are linked [29]. Different microbial communities determine different microbial functions and productions, thereby contributing to the pathogenesis and development of different diseases [37–39]. Based on LDA selection, gut microbial functions involved in Leucine and LPS biosynthesis enriched, while Spermidine putrescine transport system and Histidine biosynthesis reduced in PC. These microbial functional alterations were consistent with microbial taxonomic changes in PC. Notably, the decrease of LPS biosynthesis function was corresponding to the reduction of LPS-producing bacteria. As a pathogen-associated molecular pattern, LPS might possess the pro-inflammatory pro-tumor capacity, and could provoke an inflammatory response and thus aggravated inflammation-related chronic conditions. High levels of LPS activated the NF-kB pathway, produced pro-inflammatory cytokines (TNF-a, IL-6 and IL-1) and led to chronic inflammation and oxidative damage [40]. Long-term chronic inflammation and oxidative damage further led to the development of cancer. These findings of microbial functions may provide useful insights into the etiology of PC.

Bile acids are physiological detergents that generate bile flow and facilitate intestinal absorption and transport of lipids, nutrients, and vitamins. The gut-liver circulation of bile acids exerts important physiological functions in feedback inhibition of bile acid synthesis and in control of whole-body lipid homeostasis [41]. Thus, it is essential to explore interactions between bile acids and gut microbiota in digestive diseases. Many PC patients always accompanied with obstruction of common bile duct, thereby leading to bile deficiency in the gut. The stratification analysis of PCH indicated that gut microbial community in PCH-O were significantly different from PCH-unO. The abundances of *Streptococcus* and *Escherichia Shigella* might distinguish PCH-O from PCH-unO to some extent. Also, the abundance of *Streptococcus* significantly increased in PCH versus PCB, and might distinguish PCH from PCB with an AUC of 0.734. These results suggested that *Streptococcus* was closely associated with the bile in the gut for PC. Metabolism and biosynthesis of bile acids and lipid were disordered in liver diseases. Previous studies indicated that genus *Streptococcus* was enriched [36], and family *Streptococaceae* was positively correlated with the increasing Child-Turcotte-Pugh score [35] in liver cirrhosis. For PC patients in our study, under bile deficiency in the gut, microbial functions associated with bile acid synthesis and lipid homeostasis presented significant changes, such as the decrease of Inosine monophosphate biosynthesis, C5 isoprenoid biosynthesis non mevalonate pathway and Tyrosine biosynthesis, and the increase of Threonine biosynthesis, Sorbitol mannitol transport system and Shikimate pathway of phosphoenolpyruvate. These findings hinted that genus *Streptococcus* was associated with bile acid and lipid homeostasis in the gut.

Gut microbiota has been linked with various chronic diseases such as obesity [42], T2D [33] and liver cirrhosis [35]. Using an approach of conventionality of germ-free mice, the seminal paper that indicated a putative causality between gut microbiota and obesity [43] demonstrated that conventionalization of previously lean and insulin-sensitive germ-free mice increased their adiposity by 60% while also increasing their insulin resistance, despite the mice having a reduced food intake [20]. Subsequently, conventionalization of germ-free mice with gut microbiota from obese mice led to substantially higher adiposity than conventionalization with the microbiota from lean mice [44, 45]. Nevertheless, most of studies on gut microbiota just showed a correlated relationship between gut microbiota and the specific disease. Our study is the first time to illustrate the characteristics in clinical PC patients, and indicates gut microbial alterations in PC, which is the advantage of this study. In contrast, this study cannot illustrate whether these gut microbial changes are a cause or effect for clinical PC, which is the limitation of this study. Thus, an approach of conventionalization of germ-free mice or fecal microbial transplantation to disease animal model is essential to validate the possible causative relationship between gut microbiota and PC development. Also, an independent validation of clinical PC samples is important to further illustrate gut microbial alteration.

## MATERIALS AND METHODS

### Ethics statement

This study was approved by the Institutional Review Board of the First Affiliated Hospital, School of Medicine, Zhejiang University (reference number 2014–336), and the study was performed in accordance with the Helsinki Declaration and Rules of Good Clinical Practice. All participants approved and signed written informed consents upon enrollment.

### Participants information

Pancreatic tumor was initially diagnosed according to international guidelines by comprehensive consideration of clinical symptoms, physical signs, contrast-enhanced abdominal tomography and magnetic resonance imaging, laboratory tests and medical history. According to current standard staging procedure for pancreatic tumor, the patients underwent laparoscopic or laparotomic examination, endoscopic retrograde cholangiopancreatography (ERCP) or endoscopic ultrasonography (EUS) with fine needle aspiration or bile duct brushing, and then biopsies were obtained for histology diagnosis of the primary tumor. Exclusion criteria for patients was as follows: (a) patients with severe complications; (b) patients with previous history of chemotherapy or abdominal irradiation were considered ineligible; (c) patients suffered from other diseases including hepatic diseases, intestinal diseases, hypertension and metabolic diseases. Further inclusive criteria was: age of 30–70 years, histological adenocarcinoma, presence of measurable or evaluable lesion, no contraindications for FDG-PET-CT imaging, adequate bone marrow reserve, normal renal function, BMI > 20. Correspondingly, HC with matched age, gender and BMI were screened and enrolled. Inclusion and exclusion criteria for HC referred to our previous study [36]. All participants who received antibiotics and/or probiotics within 8 weeks before enrollment were also excluded. Patients information on diet data, drug use, drinking and so on were collected (the details in Patient information collection).

### Sample collection and DNA extraction

Stool samples of patients with pancreatic tumor were collected at 6:30–8:30 am when they were admitted to hospital. Each participant provided a fresh stool sample that was delivered immediately from our hospital to the laboratory in an ice bag using insulating polystyrene foam containers. The sample was divided into five aliquots of 200 mg and immediately stored at −80 °C. The sample that stayed in room temperature more than 2 hours was discarded. A frozen aliquot (200 mg) of each fecal sample was processed by phenol trichloromethane DNA extraction using a bead beater to mechanically disrupt cells, followed by phenol–chloroform extraction [36, 45]. DNA was further purified using the Quick gel extraction kit (Qiagen, Germany) according to the manufacturer's instructions. DNA concentration was measured by NanoDrop (Thermo Scientific), and its molecular size was estimated by agarose gel electrophoresis.

### PCR amplification and MiSeq sequencing

The extracted DNA samples were amplified with a set of primers targeting the hyper-variable V3-V5 region (338F/806R) of the 16S rRNA gene. The forward primer is 5′-ACTCCTACGGGAGGCAGCA-3′ and the reverse primer is 5′-GGACTACHVGGGTWTCTAAT-3′. The PCR amplification was performed according to our previous description [36, 46]. DNA libraries were constructed according to the manufacturer's instructions, and the sequencing was performed on the Illumina MiSeq platform at the Majorbio Bio-Pharm Technology Co., Ltd. The raw Illumina read data for all samples have been deposited in the European Bioinformatics Institute European Nucleotide Archive database under accession number PRJEB13286 (Secondary study accession number: ERP014841).

### Sequence data process

The amplified reads were processed with following steps: (a) pair end sequenced reads of each library were overlapped by FLASH version 1.2.10 [47] with default parameters. (b) a custom per program was used to perform more specific quality control of overlapped reads generated by FLASH: 1) No ambiguous bases (N) were allowed in reads; 2) No more than 5 mismatches were allowed in overlap region; 3) No mismatches were allowed in barcode/primer region. (c) reads were de-multiplexed and assigned into different samples according to barcodes; (d) chimeric sequences were detected and removed with UCHIME version 4.2.40 [48] with 16S “golden standard” database provided by Broad Institute as reference (version microbiome util-r20110519, http://drive5.com/uchime/gold.fa).

### OTUs clustering and taxonomy annotation

Random reads were chosen from all samples with equal number, and then OTUs were binned by UPARSE pipeline [49] with following steps: (a) abundant sequences and singletons were firstly removed; (b) unique sequences were binned into OTUs with command “usearch-cluster_otus”; (c) randomly chosen sequences were aligned against OTU sequences with command “usearch-usearch_global-id 0.97”, the identity threshold was set as 0.97, and then OTU composition table was created. We annotated sequences by using RDP classifier version 2.6 [50], confident level was set as 0.5 according to the developer's documents (http://rdp.cme.msu.edu/classifier/class_help.jsp#conf).

### Bacterial diversity and principal coordinate analysis (PCoA)

Bacterial diversity was determined by sampling-based analysis of OTUs, and shown by Shannon, Chao1 and Simpson indices estimated at a distance of 3% that were calculated using R program package “vegan” [51]. PCoA based on OTUs distribution was conducted by the R package (http://www.R-project.org/) to visualize interactions among bacterial communities. The weighted and unweighted unifrac distances were calculated with phyloseq package [52]. Bacterial differences at the taxonomic level including phylum, class, order, family and genus, were compared.

The specific characterization of fecal microbiota to distinguish taxonomic types was analyzed by linear discriminant analysis (LDA) effect size (LEfSe) method (http://huttenhower.sph.harvard.edu/lefse/) [53]. Using a normalized relative abundance matrix, LEfSe performs the Kruskal-Wallis rank sum test to determine the features with significantly different abundances between assigned taxa and uses LDA to assess the effect size of each feature [54].

### Functional annotation of 16S rRNA gene based on KEGG profile

The PICRUSt version 1.0.0 pipeline [29] and human version 0.99 [30] were used to construct KEGG orthology (KO) and KEGG pathway/module profile, predicting functional profiling of microbial communities using 16S rRNA marker gene sequences. PICRUSt uses an extended ancestral-state reconstruction algorithm to recaptures key findings from the Human Microbiome Project and accurately predicts gene families abundance in host-associated communities, with quantifiable uncertainty.

### Probability of disease (POD) calculation

First we built 10-fold cross validation model by using R package “randomForest” [31]. For a validation sample, models were trained by using unrelated train samples. POD is calculated as follows:

$$\text{POD}=\frac{D}{M}$$

M is the number of all trees (this was set as 1000 in our study), and tree D is the number of trees that will classify validation sample as “disease” status. For HC versus PC, we term PC as disease. For PCH-O versus PCH-unO, we term PCH_O as disease.

### Statistical analysis

Continuous variables were compared using Wilcoxon rank sum test between both groups. One-way ANOVA was utilized to evaluate difference among three groups. Fisher's exact test was used to compare categorical variables. Receiver operating characteristics (ROC) curves were conducted and AUC was used to designate ROC effect. Statistical analyses were performed using SPSS version 19.0 for Windows (SPSS Inc., Chicago, IL).

## CONCLUSIONS

To our knowledge, this is the first report to illustrate gut microbial characteristics in PC through a large-cohort Miseq sequencing. The patients with PC presented a decreased diversity of gut microbiota, and an unique microbial profile distinguishing from HC, partly attributed to the decrease of alpha diversity. Gut microbial alterations in PC showed an increase of some potential pathogens and LPS-producing bacteria, and a decrease of some probiotics and butyrate-producing bacteria. The alterations of microbial gene functions were consistent with taxonomic changes in PC. *Streptococcus* was associated with the bile in the gut. Based on crucial genera associated with PC, microbial markers might achieve a high classification power for PC. These findings may provide non-invasive bio-markers for PC diagnosis.

### Availability of data and materials

The raw Illumina read data for all samples have been deposited in the European Bioinformatics Institute European Nucleotide Archive database under accession number PRJEB13286 (Secondary study accession number: ERP014841).

### Consent for publication

Consent was not required as data are anonymized.

### Ethics approval and consent to participate

This study was approved by the Institutional Review Board of the First Affiliated Hospital, School of Medicine, Zhejiang University (reference number 2014–336), and the study was performed in accordance with the Helsinki Declaration and Rules of Good Clinical Practice. All participants approved and signed written informed consents upon enrolment.

## SUPPLEMENTARY MATERIALS FIGURES AND TABLES















## Abbreviations

PC: pancreatic carcinoma
HC: healthy controls
PCH: pancreatic head carcinoma
PCB: pancreatic body and tail carcinoma
PCH-O: PCH with obstruction of common bile duct
PCH-unO: PCH with un-obstruction of common bile duct
BMI: body mass index
DB: direct bilirubin
ALT: alanine transaminase
AST: aspartate aminotransferase
OTUs: Operational Taxonomy Units
PCoA: principal coordinate analysis
LDA: linear discriminant analysis
LEfSe: LDA effect size
LPS: lipopolysaccharides
AUROC: area under the receiver operating characteristics curve
AUC: area under the curve
95% CI 95%: confidence interval
PICRUSt: phylogenetic investigation of communities by reconstruction of unobserved states
POD: probability of disease
T2D: type 2 diabetes
ROC: receiver operating characteristics.
