# Supplementary material for: Gut microbial profile analysis by MiSeq sequencing of pancreatic carcinoma patients in China
Source: Oncotarget. 2017 Jun 29;8(56):95176–91. doi: 10.18632/oncotarget.18820 (PMC5707014; doi:10.18632/oncotarget.18820)
Supplement: Supplementary file 1 [file oncotarget-08-95176-s001.pdf]

Gut microbial profile analysis by MiSeq sequencing of pancreatic carcinoma patients in China

Supplementary Materials

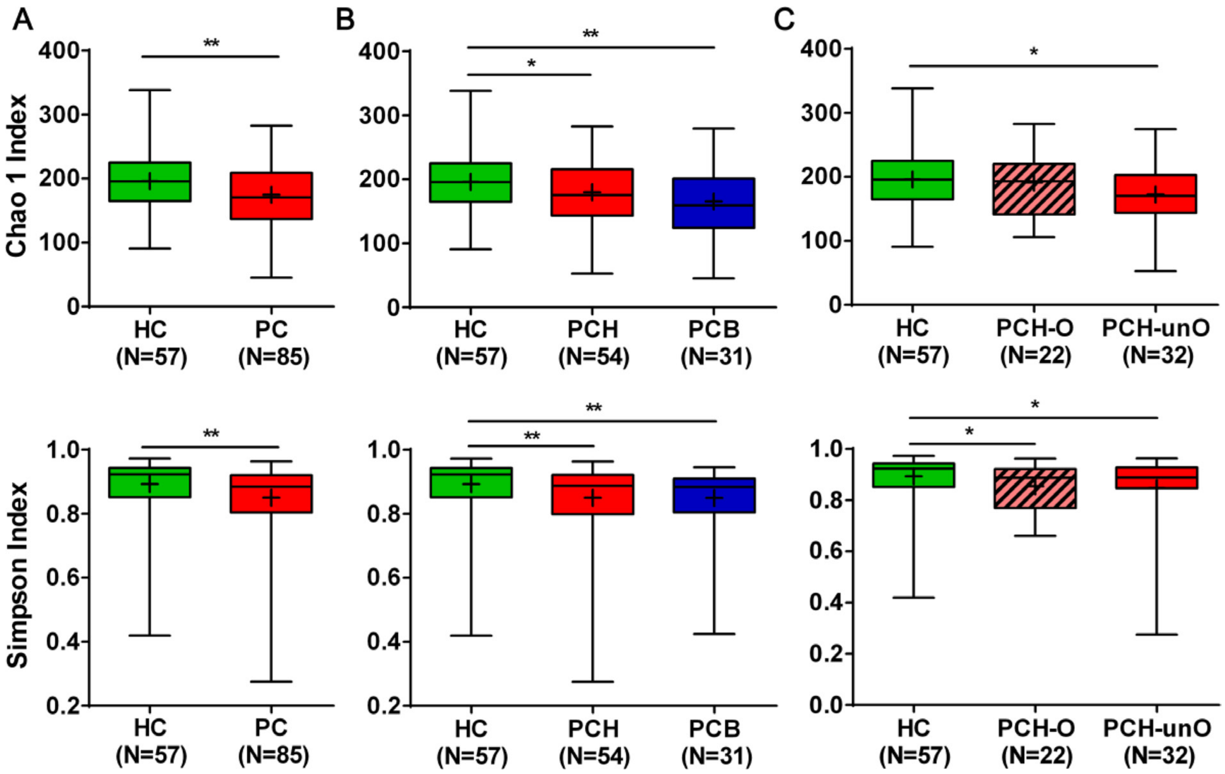

Supplementary Figure 1: Microbial alpha diversity is decreased in PC patients, shown by the Chao 1 and Simpson diversity indices. (**A**) The comparison of the Chao 1 and Simpson diversity indices between PC patients (n = 85) and HC (n = 57). (**B**) The subgroup comparison of the Chao 1 and Simpson diversity indices among HC (n = 57), PCH (n = 54) and PCB (n = 31). (**C**) The stratification analysis of the Chao 1 and Simpson diversity indices among HC (n = 57), PCH-O (n = 22) and PCH-unO (n = 32). The box presented the interquartile range; the line inside denotes the median, and the symbol "+" denotes the mean value. HC, healthy controls; PC, pancreatic carcinoma; PCH, PC (head); PCB, PC (body and tail); PCH-O, PCH patients with obstruction of bile duct; PCH-unO, PCH patients with unobstruction of bile duct. *$p < 0.05$, **$p < 0.01$.

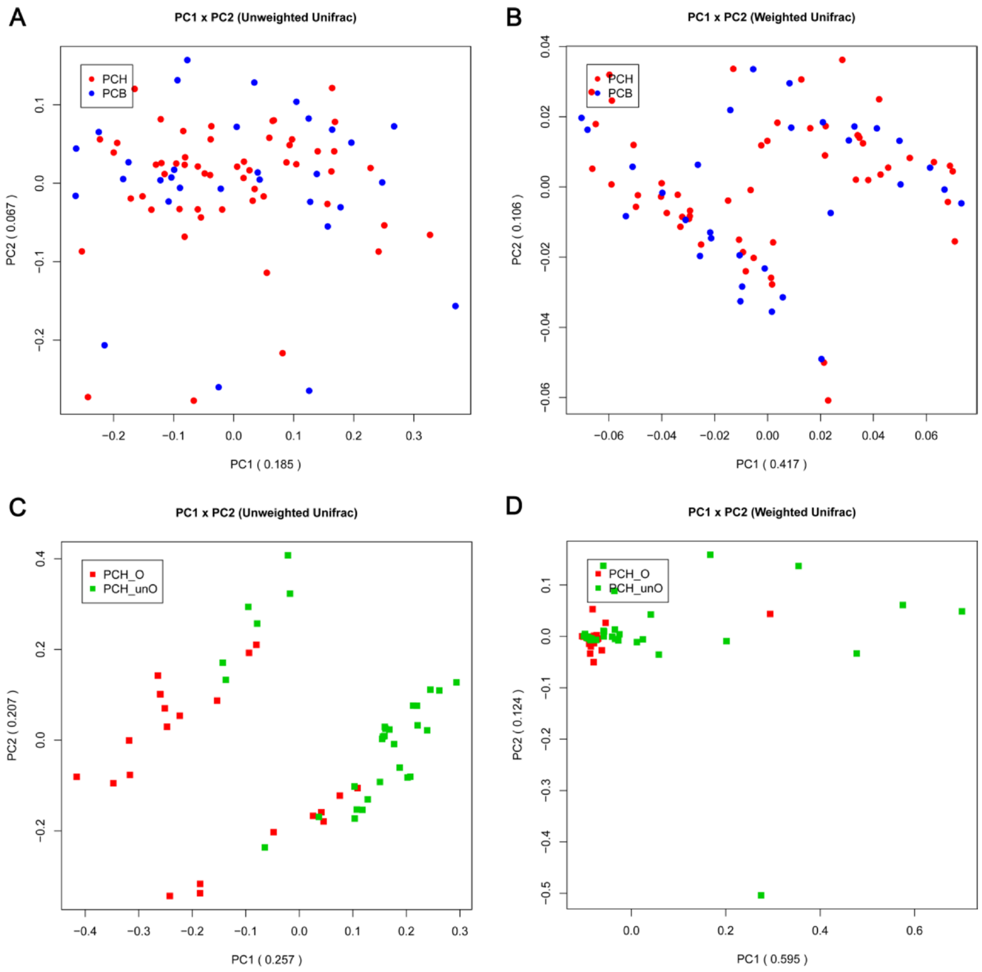

Supplementary Figure 2: Principal coordinate analysis (PCoA) of gut microbial community for the subgroup analysis and stratification analysis of PC patients. PCoA of gut microbial community for PCH patients and PCB patients in the unweighted Unifrac plot (**A**) from PC1 and PC2 (18.5% and 6.7%) and the weighted Unifrac plot (**B**) from PC1 and PC2 (41.7% and 10.6%). PCoA of gut microbial community for PCH-O patients and PCH-unO patients in the unweighted Unifrac plot (**C**) from PC1 and PC2 (25.7% and 20.7%, $p < 0.001$) and the weighted Unifrac plot (**D**) from PC1 and PC2 (59.5% and 12.4%, $p < 0.001$).

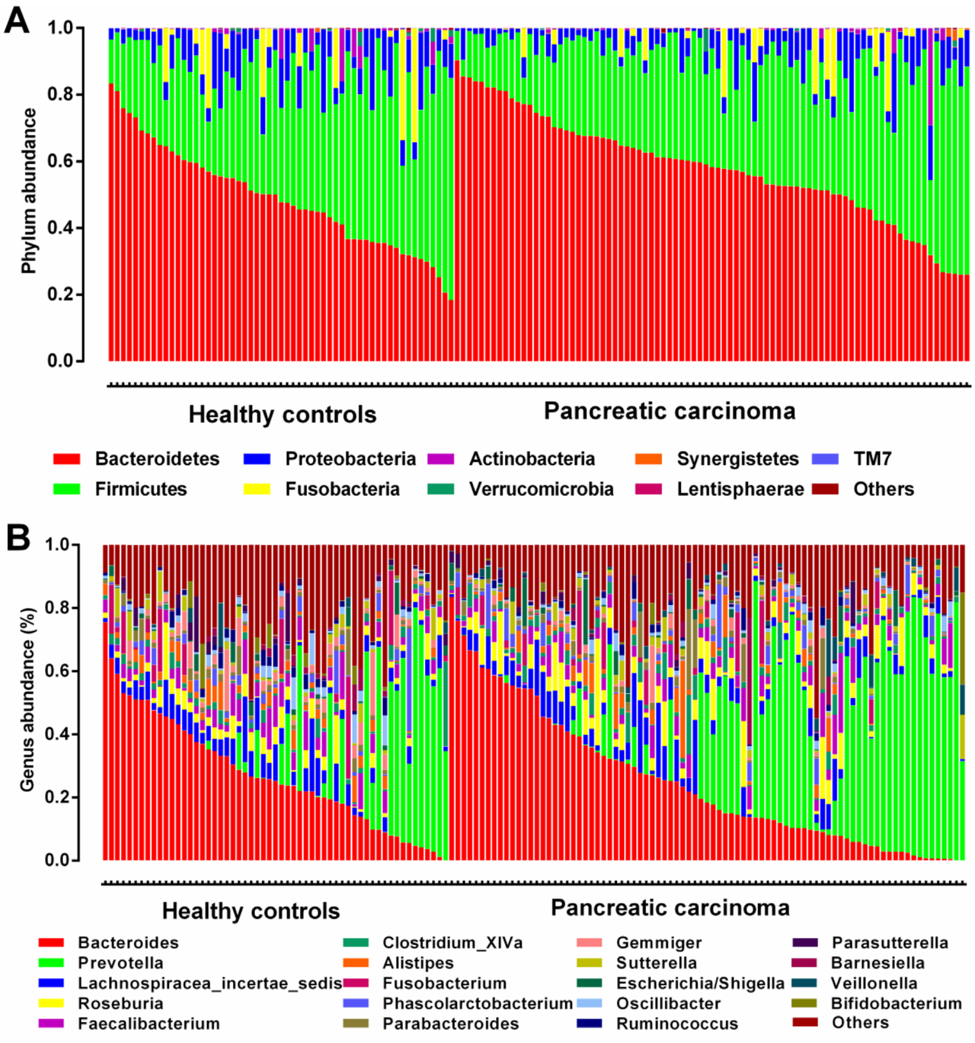

Supplementary Figure 3: Composition and distribution of fecal microbial communities at the phylum and genus levels among all samples (*n* = 142). (**A**) Composition and distribution of fecal microbial communities at the phylum level among all samples (*n* = 142). The top 9 bacterial populations were shown in all samples. (**B**) Composition and distribution of fecal microbial communities at the genus level among all samples (*n* = 142). The top 19 bacteria at the genus level were listed in all samples.

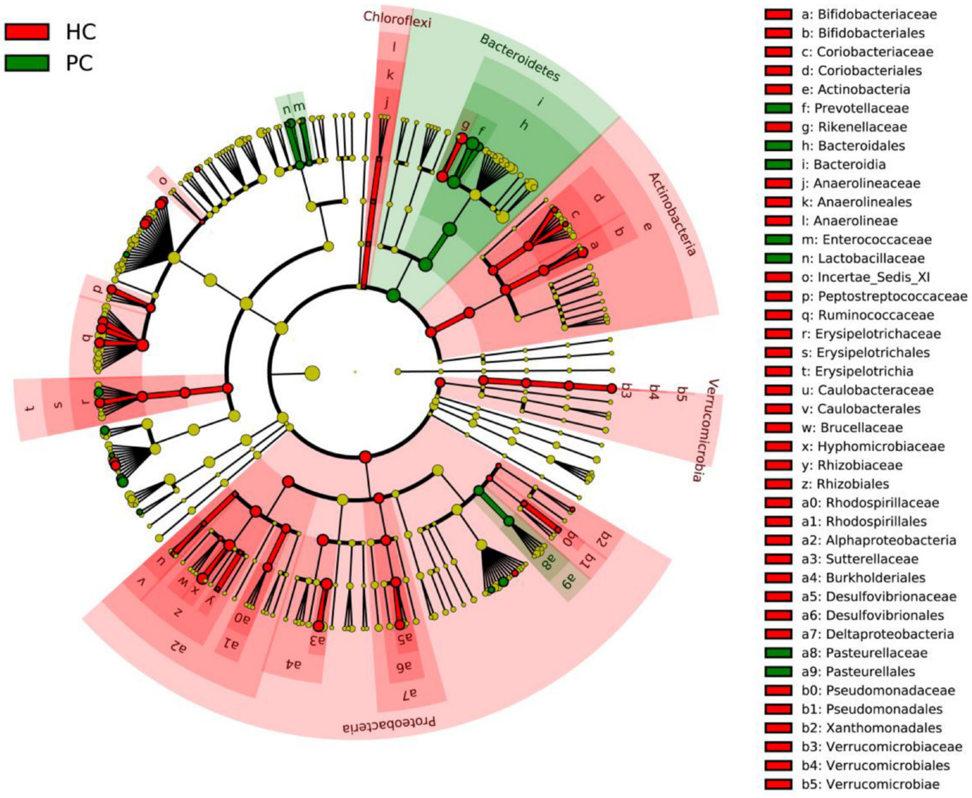

Supplementary Figure 4: Phylogenetic profiles of the specific bacterial taxa and the predominant bacteria between PC patients (*n* = 85) and healthy controls (*n* = 57) using the LEfSe method. The specific characterization of fecal microbiota to distinguish toxigenic types was analyzed by linear discriminant analysis (LDA) effect size (LEfSe) method (http://huttenhower.sph.harvard.edu/lefse/).

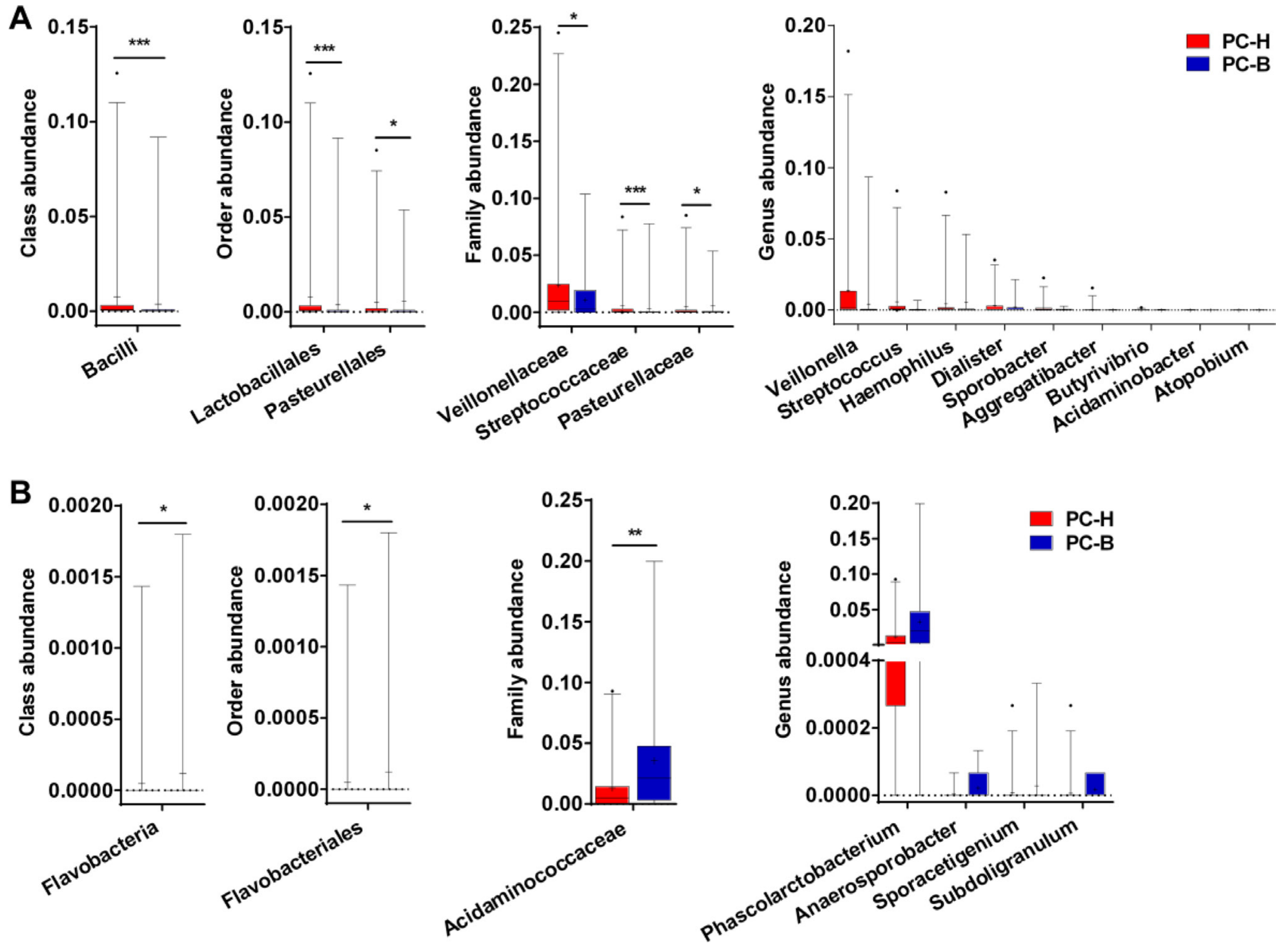

Supplementary Figure 5: Difference of fecal microbial communities at the taxonomic levels between PCH patients (*n* = 54) and PCB patients (*n* = 31). (**A**) The increased bacterial populations at the class, order, family and genus levels in the PCH patients, respectively, as compared with the PCB patients. (**B**) The decreased bacterial populations at the class, order, family and genus levels in the PCH patients, respectively, as compared to the PCB patients. The box presented the interquartile range; the line inside denotes the median, and the symbol "+" denotes the mean value. PCH, pancreatic carcinoma (head); PCB, pancreatic carcinoma (body and tail).

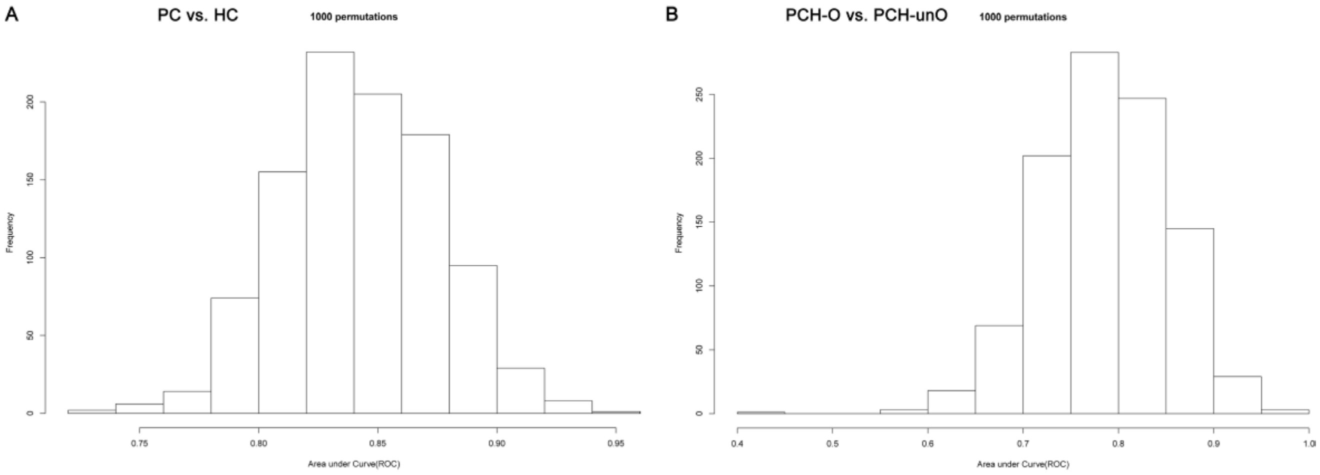

Supplementary Figure 6: Histogram of AUC values of combined classification of the crucial bacteria using LDA selection based on 1000 permutations. (**A**) Histogram of AUC values of the 40 genera associated with PC using LDA selection based on 1000 permutations. (**B**) Histogram of AUC values of the 10 genera associated with PCH-O using LDA selection based on 1000 permutations. H, healthy controls; PC-B, pancreatic carcinoma (body and tail). HC, healthy controls; PC, pancreatic carcinoma; PCH, PC (head); PCH-O, PCH patients with obstruction of bile duct; PCH-unO, PCH patients with unobstruction of bile duct.

Supplementary Data File 1: A total of 880 Operational Taxonomy Units (OTUs) were obtained and annotated. See Supplementary_Data_File_1

Supplementary Data File 2: The relative values of the 535 qualified OTUs were shown between pancreatic carcinoma and healthy controls. See Supplementary_Data_File_2

Supplementary Data File 3: Gut microbial diversity indices were evaluated by Shannon index, Simpson index, Invsimpson index, OBS index, Chao 1 index and ICE index between pancreatic carcinoma and healthy controls. See Supplementary_Data_File_3

Supplementary Data File 4: Phylogenetic investigation of communities by reconstruction of unobserved states (PICRUSt) version 1.0.0 pipeline and human version 0.99 were utilized to construct KEGG pathway/module profile and predict microbial functional capacity of microbial communities using 16S rRNA marker gene sequences. See Supplementary_Data_File_4

Supplementary Data File 5: Gut microbial gene function prediction between pancreatic carcinoma and healthy controls based on linear discriminant analysis (LDA) selection. See Supplementary_Data_File_5

Supplementary Data File 6: Gut microbial gene function prediction between pancreatic carcinoma (head) with obstruction and unobstruction of common bile duct based on linear discriminant analysis (LDA) selection. See Supplementary_Data_File_6
